# Inflammatory cytokines, goblet cell hyperplasia and altered lung mechanics in *Lgl1*^+/- ^mice

**DOI:** 10.1186/1465-9921-10-83

**Published:** 2009-09-21

**Authors:** Jie Lan, Leslie Ribeiro, Isabel Mandeville, Katia Nadeau, Tim Bao, Salomon Cornejo, Neil B Sweezey, Feige Kaplan

**Affiliations:** 1 McGill University - Montreal Children's Hospital Research Institute Montreal, Quebec, H3Z2Z3, Canada; 2 Department of Human Genetics, McGill University, Montreal, Quebec H3A1B1, Canada; 3 Department of Biology, McGill University Montreal, Quebec H3A1B1, Canada; 4 Hospital for Sick Children Research Institute, Toronto, Ontario M5G 1X8, Canada; 5 Departments of Pediatrics and Physiology, University of Toronto, Toronto, Ontario, Canada; 6 Department of Pediatrics, McGill University, Montreal, Quebec, Canada

## Abstract

**Background:**

Neonatal lung injury, a leading cause of morbidity in prematurely born infants, has been associated with arrested alveolar development and is often accompanied by goblet cell hyperplasia. Genes that regulate alveolarization and inflammation are likely to contribute to susceptibility to neonatal lung injury. We previously cloned *Lgl1*, a developmentally regulated secreted glycoprotein in the lung. In rat, O_2 _toxicity caused reduced levels of *Lgl1*, which normalized during recovery. We report here on the generation of an *Lgl1 *knockout mouse in order to determine whether deficiency of *Lgl1 *is associated with arrested alveolarization and contributes to neonatal lung injury.

**Methods:**

An *Lgl1 *knockout mouse was generated by introduction of a neomycin cassette in exon 2 of the *Lgl1 *gene. To evaluate the pulmonary phenotype of *Lgl1*^+/- ^mice, we assessed lung morphology, *Lgl1 *RNA and protein, elastin fibers and lung function. We also analyzed tracheal goblet cells, and expression of mucin, interleukin (IL)-4 and IL-13 as markers of inflammation.

**Results:**

Absence of *Lgl1 *was lethal prior to lung formation. Postnatal *Lgl1*^+/- ^lungs displayed delayed histological maturation, goblet cell hyperplasia, fragmented elastin fibers, and elevated expression of T_H_2 cytokines (IL-4 and IL-13). At one month of age, reduced expression of *Lgl1 *was associated with elevated tropoelastin expression and altered pulmonary mechanics.

**Conclusion:**

Our findings confirm that *Lgl1 *is essential for viability and is required for developmental processes that precede lung formation. *Lgl1*^+/- ^mice display a complex phenotype characterized by delayed histological maturation, features of inflammation in the post-natal period and altered lung mechanics at maturity. *Lgl1 *haploinsufficiency may contribute to lung disease in prematurity and to increased risk for late-onset respiratory disease.

## Background

Impaired alveolar development is a leading cause of neonatal morbidity in premature infants weighing less than one kilogram. Deficient alveolar maturation in these children is often characterized by distal airspace enlargement, disruption of elastin fibers and mucus cell hyperplasia. Antenatal exposures of the premature lung may increase susceptibility to inflammation and subsequent postnatal (PN) lung injury. Genes that regulate alveolarization, innate immunity and inflammation are likely to contribute to susceptibility to and outcome in neonatal lung disease.

In a search for downstream targets of glucocorticoid (GC) that regulate lung maturation, we cloned *Lgl1 *(late gestation lung 1), a developmentally regulated gene in the lung [1-3]. *Lgl1 *is a CRISP family (cystine rich secretory protein) protein characterized by a secretory signal and two LCCL (also known as FCH) domains [4-6]. The LCCL domain, an as yet poorly understood module found in over 100 extracellular proteins, has been implicated in directional cell migration and differentiation [5,7], extracellular matrix deposition [5,8], cell adhesion [9] and innate host-defense mechanisms [4,10,11]. While *Lgl1 *synthesis is almost exclusively restricted to the mesenchyme, *Lgl1 *protein is associated with lung epithelial cells from the late canalicular period onward [2,3]. We showed previously that *Lgl1 *protein stimulates airway branching in lung explant culture [12]. Maximal fetal expression of *Lgl1 *was, however, concordant with the onset of augmented surfactant production in late gestation [1,3]. In postnatal rat lung, *Lgl1 *concentrated at the tips of budding alveolar septa [3]. Levels of *Lgl1 *were drastically reduced in rat O_2 _toxicity models of bronchopulmonary dysplasia (BPD), a chronic lung disease of impaired alveolarization in premature infants, and were restored during recovery in air [3]. Taken together, these observations suggested that *Lgl1 *may regulate both early and late events in lung organogenesis.

We now report on the development of an *Lgl1 *knockout mouse to investigate the *in vivo *function of *Lgl1 *in regulating multiple aspects of lung development. Absence of *Lgl1 *in homozygous null (*Lgl1*^-/-^) mice was not compatible with life. We describe here the lung phenotype of *Lgl1*^+/- ^heterozygous mice. Lungs of developing *Lgl1*^+/- ^mice were characterized by disorganized elastin fibers, early expression of inflammatory cytokines and goblet cell hyperplasia. In mature *Lgl1*^+/- ^mice, reduced *Lgl1 *expression was associated with altered lung mechanics.

## Methods

### Generation of Lgl1 knockout mice

All procedures involving animals were conducted according to criteria established by the Canadian Council for Animal Care and approved by the Animal Care Committee of the McGill University Health Centre. A 13.7 kb EcoR1 fragment of BAC clone 34304 containing the *Lgl1 *gene was subcloned into pQZ1BamH1 and a Neo cassette was used to replace exon 2. Not1-Sal1 digestion of this construct produced a 9.6 kb targeting fragment that was electroporated into ES cells. Southern analysis and PCR were used to verify accuracy of targeting. Mouse genotypes were verified by PCR. The primers used were (5'-3'): reverse (wild type) CACTGCTCCGTGTATCAAGCATACAC; reverse (NeoI) GACAATCG GCTGCTCTGATG; or reverse (5' to3') TCGTCGTGACCCATGGCGAT (NeoII) and forward (for all 3 reactions) CAGGTCTGGCTCTGAGGTTCTTGCA. The expected amplification products were: 0.8 kb (wild type), 1 kb (Neo1) and 0.46 kb (Neo2). For details on mouse preparation see Additional file 1.

### Isolation of Total Lung RNA

Total RNA was prepared from lungs, brain, heart, kidney, thymus and spleen using Trizol reagent (Invitrogen, Burlington, ON, Canada) according to the manufacturer and was resuspended in 1× RNASecure (Ambion, Austin, TX, USA). RNA was pooled for each litter according to genotype (N ≥ 4).

### Quantitative Real-Time RT-PCR

Quantitative real-time RT-PCR was performed on the Mx4000 QPCR system (Stratagene, La Jolla, CA, USA) using the Quantitect One-Step Probe RT-PCR Kit (Qiagen, Mississauga, ON, Canada) as directed by supplier. Gene-specific primers and FAM labeled probes for mouse *LGL1*, IL-4, IL-13, Mucin5AC and Tropoelastin were designed using Qiagen's online QuantiProbe Design Software. Quantitect Gene Expression Assay for mouse 18S (Qiagen, Mississauga, ON, Canada) was used to normalize for the input of RNA. The results were analyzed according to the standard curve method. One-step real-time RT-PCR reactions were performed in 25 μL volumes for 40 cycles, using 20 ng of total RNA for *Lgl1*, IL-4, IL-13, Mucin5AC and Tropoelastin and 50 pg for 18S. For a list of primers and probes used see Additional file 2.

### Bronchoalveolar Lavage (BAL)

BAL was performed by instilling the lungs four times with 1 ml cold phosphate-buffer saline through a tracheal cannula. Lavage fluid was centrifuged and pellets were resuspended in 0.5 ml cold saline. Total cell numbers were counted with a haemocytometer. For differential cell counts, cytospin slides were prepared (Cytospin 4; Shandon, Pittsburgh, PA) and stained with Diff-Quick; at least 200 cells/slide were counted and percentage of each cell type was calculated.

### Lung fixation

Lungs were inflated with 4% paraformaldehyde at a pressure of 20 cm of water. Lungs were gently extracted and fixed in 4% paraformaldehyde overnight. Samples were dehydrated through a series of increasing ethanol washes and embedded in paraffin. 5 μm thick tissue slices were cut through the entire lung.

### Antibodies

*Lgl1*, 1:100 (Covance, Quebec, Canada), β-actin, 1:5000 (Sigma-Aldrich, Oakville, ON, Canada), Anti-Rabbit IgG HRP conjugated, 1:5000 (Amersham, Little Chalfont, Buckinhamshire, UK), Anti-Rabbit IgG AlexaFluor 594 and Anti-Rat IgG AlexaFluor 488 conjugated, 1:200 (Invitrogen, Burlington, ON, Canada), CD34, 1:100 (Abcam, Cambridge, MA, USA).

### Immunohistochemistry

Sections of paraffin-embedded lung tissue were stained with hematoxylin and eosin or used for histochemical staining. Ten sections from each of at least 4 and up to 7 animals were assessed for all histochemical experiments and representative images shown in all figures. For immunohistochemistry, slides were rehydrated through a series of decreasing ethanol washes, rinsed with PBS-0.03% Triton and incubated in warm 10 mM sodium citrate for antigen retrieval. Slides were then incubated in H_2_O_2 _and methanol for 20 minutes to block endogenous peroxidase activity. To block non-specific binding, slides were incubated in PBS-0.03% Triton containing 5% normal goat serum and 1% BSA. Primary antibodies were incubated overnight at 4°C and the following day in corresponding fluorescent conjugated secondary antibody for 30 minutes at room temperature. For CD34 and *Lgl1 *co-immunohistochemistry, the slides were then washed with PBS-0.03% Triton and blocked again with 5% normal goat serum and 1% BSA. The second primary antibody was incubated overnight at 4°C and the following day in corresponding fluorescent conjugated secondary antibody for 30 minutes at room temperature. Slides were washed with PBS-0.03% Triton and mounted with pro-long anti-fade media containing DAPI (Invitrogen, Burlington, ON, Canada). For the co-staining, the DAPI is not shown to improve visualization. For *Lgl1 *immunohistochemistry, protein levels were quantified using the Northern Eclipse program (Northern Eclipse software, Empix Imaging, Inc. Mississauga, ON, Canada) as per Nadeau *et al. *2006 [3].

### Identification of Goblet cells

Slides were rehydrated through a series of decreasing ethanol washes and stained with Periodic Acid-Schiff kit (Sigma-Aldrich, Oakville, ON, Canada) to visualize goblet cells. For goblet cell staining, sections from at least 6 and up to 10 animals were assessed and representative images shown in all figures.

### Elastin Staining

Slides were rehydrated through a series of decreasing ethanol washes and elastin fibers were stained with Fuschin Weigert stain and counterstained with methyl green for better visualization. For elastin staining sections from at least 4 and up to 7 animals were assessed and representative images shown in all figures.

### Morphometry

Mouse lungs were fixed under constant distending pressure of 20 cm of fixative. Morphometric measurements were made on hematoxylin and eosin stained lung sections (n > 5 mice). A minimum of 10 representative fields were studied in each lung. A computer generated grid (384 intersections) was superimposed on digital images and grid intersections were examined to determine whether they localized to airspace or tissue. Percent fractional airspace or fractional area of lung parenchyma was quantified using Northern Eclipse software.

### Lung Mechanics

At 4 weeks of age, mice were deeply anaesthetized by an i.p. injection of xylazine (8 mg/kg) and pentobarbital (70 mg/kg), tracheotomized and placed on a small animal ventilator (flexiVent, SCIREQ, Canada). Animals were ventilated quasi-sinusoidally (150 breaths/min and tidal volume of 10 ml/kg) and subsequently paralyzed by an i.p. injection of pancuronium bromide (0.8 mg/kg). Maximal resistance and elastance were recorded before and after increasing doses of aerosolized methacholine.

### Statistical Analysis

All results are expressed as mean ± standard error of the mean. P ≤ 0.05 was considered to be statistically significant as measured by student t-test or ANOVA as appropriate.

The numbers of goblet cells in postnatal day 14 lungs displayed a single modal distribution in wild type, but a marked bimodal distribution in heterozygous (*Lgl1*^+/-^) mice. Subgroup analyses of the heterozygous mice revealed the data were not normally distributed; a Mann-Whitney U test showed a significant difference between the subgroups (p < 0.02).

## Results

### Absence of Lgl1 is associated with embryonic lethality

An *Lgl1 *knockout mouse was generated by introduction of a neomycin cassette in exon 2 of the *Lgl1 *gene. Analysis of progeny (79 litters, 133 wild type and 290 heterozygotes) revealed no homozygous *Lgl1*^-/- ^progeny or resorption sites (from embryonic day (E) 9.5 until birth).

We explored the lung phenotype of *Lgl1*^+/- ^heterozygotes from E14.5 until maturity at postnatal day (PN) 28.

### Lgl1^+/- ^mice have altered lung morphology

**Figure 1:**
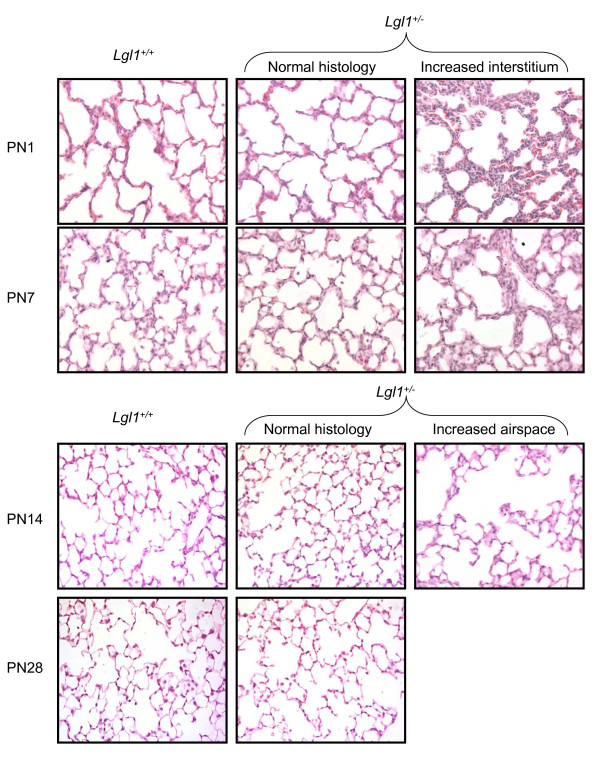
**Lgl1^+/- ^mice display altered lung morphology**. Representative micrographs of hematoxylin and eosin stained lung sections showing areas of increased interstitial tissue in *Lgl1*^+/- ^mice at PN1 and PN7, and enlarged airspace with fewer alveolar septa at PN14. For *Lgl1*^+/- ^mice, representative images of regions of impaired lung morphology are shown together with images of regions indistinguishable from those of wild type lungs. Magnification: 200×.

Heterozygous (*Lgl1*^+/-^) mice appeared normal at birth and exhibited no obvious changes in gross morphology. Lung and body weight and lung to body weight ratios were normal. On morphometric analysis, the ratio of respiratory tissue to airspace throughout the entire lung was significantly increased in *Lgl1*^+/- ^mice at PN1 (tissue fraction [39.0 ± 3.51] % *vs *[23.48 ± 2.46] % in wild type, p < 0.04). Visual inspection of the histological sections revealed that this difference was distributed unevenly, with patchy areas of distinctly thickened respiratory interstitium alternating with areas of relatively normal appearance (Figure 1). No such patches were seen in wild type lungs. With advancing PN age, distinct areas with thickened interstitium could still be identified in the lungs of heterozygote (but not wild type) mice to a diminishing degree; however, the observed trends towards an increased tissue to airspace ratio for the entire lung no longer reached statistical significance. At PN14, when lungs of wild type animals showed advanced alveolarization, lungs of *Lgl1*^+/- ^mice appeared to be at an earlier, more active phase of secondary septation with fewer alveolar secondary septa and relatively enlarged distal airspaces. By PN28, the lung morphology of *Lgl1*^+/- ^mice was indistinguishable from that of wildtype littermates.

### Lgl1 mRNA and protein expression in Lgl1^+/- ^mice

**Figure 2:**
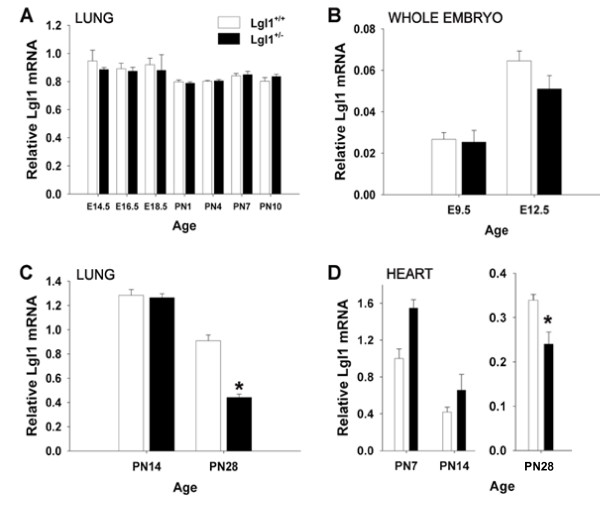
**Lgl1 mRNA is reduced in the lungs of Lgl1^+/- ^mice**. *Lgl1 *mRNA isolated from total lungs (A and C) or whole embryos (B) of *Lgl1*^+/+ ^and *Lgl1*^+/- ^mice was quantified by quantitative real-time RT PCR. A. and B. No significant differences in lung *Lgl1 *mRNA was observed from E9.5 until PN14 (p > 0.05). C. At PN28, there was significantly less *Lgl1 *mRNA in the lungs of *Lgl1*^+/- ^mice compared to their wild type littermates (p ≤ 0.05). D. Mature *Lgl1*^+/- ^mice display significantly reduced levels of *Lgl1 *mRNA in the heart when compared to their wild type littermates (p ≤ 0.05). (A. N ≥ 3 pooled litters, B. N ≥ 4, C. N ≥ 5, D. N ≥ 4)

Given the observed morphological changes, we expected that PN *Lgl1*^+/- ^mice would display aberrant expression of *Lgl1 *mRNA and *Lgl1 *protein. We used real-time quantitative RT-PCR to compare lung *Lgl1 *mRNA levels in *Lgl1*^+/- ^and *Lgl1*^+/+ ^mice (Figure 2). No significant differences were observed in *Lgl1 *mRNA in the lungs of *Lgl1*^+/-^mice compared to controls during the course of lung development from E9.5 until PN14 (Figure 2A, B). By contrast, at 4 weeks of postnatal age, when lung development in the mouse is essentially complete (alveolarization occurs mainly between PN1-PN14), a significant reduction in *Lgl1 *mRNA (~ 50%) was observed (Figure 2C). Given the absence of significantly altered *Lgl1 *expression in the lungs of developing *Lgl1*^+/-^mice, we considered the possibility that effects on lung morphology were indirect and resulted from aberrant *Lgl1 *expression in other organs. No significant differences in *Lgl1 *mRNA expression were observed in developing heart, brain, kidney, spleen and thymus (not shown). Mature *Lgl1*^+/-^mice, however, showed a limited but significant reduction in *Lgl1 *mRNA expression in the heart-concordant with the observed changes in lung *Lgl1 *mRNA expression (Figure 2D). These findings suggest that aberrant expression of *Lgl1 *in *Lgl1*^+/-^mice is tissue- and temporal-specific and may depend on availability of local and circulating regulatory factors.

**Figure 3:**
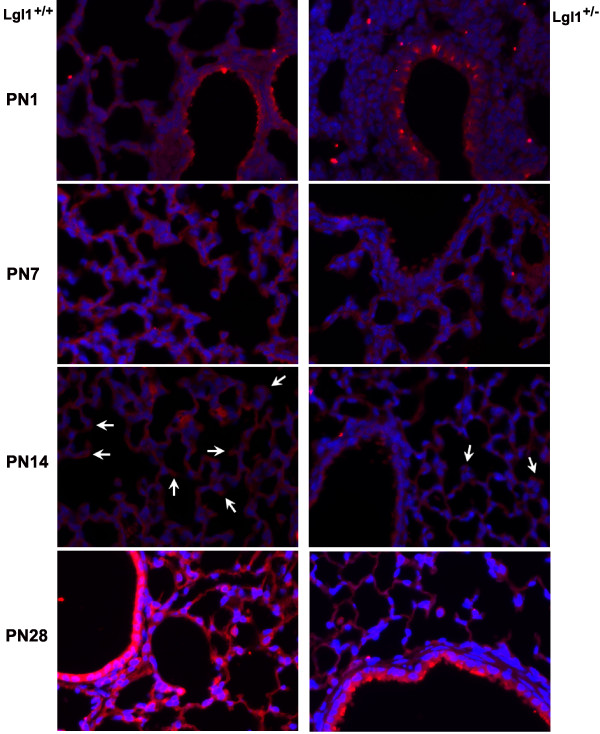
**Lgl1 protein is reduced in Lgl1^+/-^mice**. *Lgl1 *immunohistochemistry (N ≥ 4, all groups) was performed as described in Methods and quantified with Northern Eclipse as per Nadeau *et al*. 2006 [3]. Representative images are shown. Modest to moderate differences in *Lgl1 *protein levels were observed between *Lgl1*^+/+ ^and *Lgl*^+/- ^mice at ages PN1, 7 and 14 (Arrows indicate the reduction of *Lgl1 *at tips of alveolar septa in *Lgl1*^+/- ^mice at PN 14). At PN28, there was considerable variability between *Lgl1*^+/- ^mouse lung samples. In some cases, *Lgl1 *protein appeared to be reduced, consistent with the observed reduction in *Lgl1 *mRNA (shown) while in others, effects on protein expression were not detectible. Magnification: 400×

We next assessed whether variance in *Lgl1 *mRNA would be reflected in coordinate changes in levels and/or distribution of *Lgl1 *protein. Several *Lgl1 *antibodies were prepared but none consistently detected *Lgl1 *on Western blots. *Lgl1 *protein was therefore analyzed by immunohistochemistry (IHC). Representative images of *Lgl1 *IHC (n ≥ 4) are illustrated in Figure 3. Northern Eclipse software was used to quantify *Lgl1 *immunostaining. From PN7 onward, lungs of *Lgl1*^+/-^mice appeared to have reduced levels of *Lgl1 *protein, most markedly at PN28. There was considerable variability among pups. In previous studies, we showed that *Lgl1 *protein concentrated at the tips of septating alveoli in PN7 rat lung[3]. In PN7 mice, *Lgl1 *appeared to be more widely distributed in the lung and accumulation at septal tips was more clearly noted at PN14. In *Lgl1*^+/- ^mice, a reduction in *Lgl1 *protein at the tips of alveolar septa was observed at PN14 (Figure 3, see arrows).

### Lgl1 protein does not localize to PN pulmonary endothelial cells

**Figure 4:**
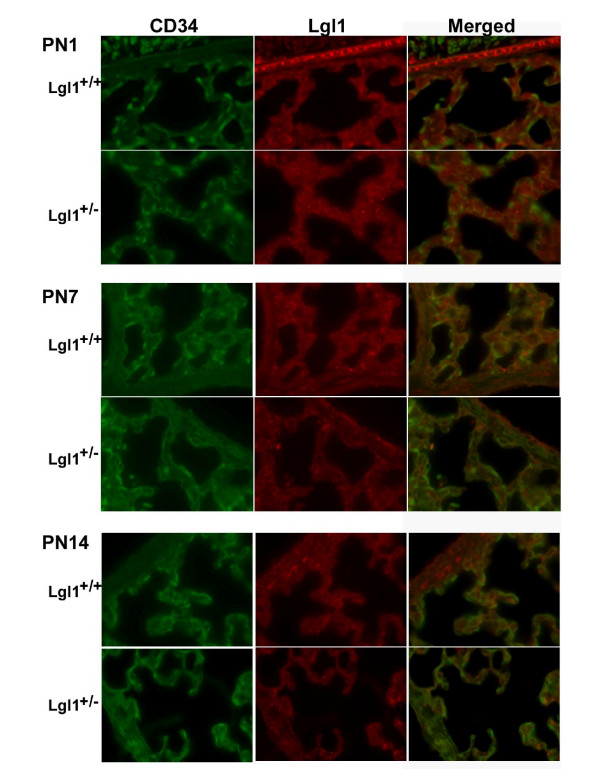
**Lgl1 does not localize to lung endothelial cells**. Immunohistochemistry for *Lgl1 *(red color) and the endothelial marker CD34 (green color; N ≥ 4, all groups) was performed as described in Methods. Representative images are shown. No colocalization (would have shown yellow colour) of *Lgl1 *and CD34 was detected in the lungs of *Lgl1*^+/+ ^or *Lgl*^+/- ^mice at ages PN1, 7 and 14. Magnification: 630×

Lung alveoli are lined by specialized Type 1 and Type 2 epithelial cells and are vascularised by an extensive capillary bed[13]. The developing lung mesenchyme undergoes vasculogenesis and angiogenesis. The eventual juxtaposition of Type 1 cells with pulmonary endothelial cells is required to facilitate gas exchange. Consistent with previous findings in rat lung,*Lgl1 *localized to both mesenchyme and epithelium in PN murine lung with high concentrations noted in epithelium surrounding the larger airways (Figure 3). To assess whether *Lgl1 *is present in endothelial cells, we evaluated colocalization of *Lgl1 *immunoreactivity (Figure 4, red color) with the endothelial marker CD34 (green color) in PN1-PN14 lung sections prepared from wild type and *Lgl1*^+/- ^mice. Representative images are shown in Figure 4 (n ≥ 4). No evidence of colocalization, which would appear as yellow color in merged images, was observed.

### Abnormal pulmonary mechanics in methacholine challenged Lgl1^+/- ^mice

**Figure 5:**
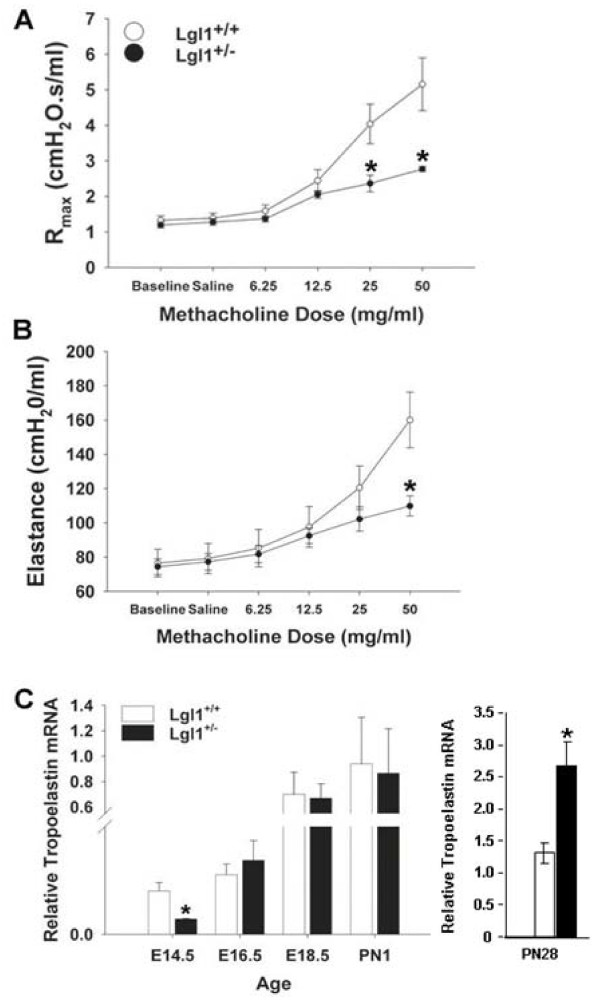
**Lgl1^+/- ^mice display reduced resistance and elastance in response to MCh challenge**. Airway hyperresponsiveness (AHR) in response to increasing doses of aerosolized MCh was assessed in 4 week old *Lgl1*^+/+ ^and *Lgl1*^+/- ^mice (N = 13 and 9 respectively). Response was measured as maximal resistance (A) and elastance (B). Means are presented ± SEMs. A. *Lgl1*^+/- ^mice displayed similar resistance to their wild type littermates at baseline. At doses of MCh ranging from 25 to 50 mg/ml *Lgl1*^+/- ^mice showed reduced resistance compared to wild type littermates. (p ≤ 0.05). B. *Lgl1*^+/- ^mice displayed similar elastance to their wild type littermates at baseline. In the presence of 50 mg/ml MCh, *Lgl1*^+/- ^mice displayed significantly less elastance than their wild type littermates (p ≤ 0.05). C. Tropoelastin mRNA isolated from total lungs of *Lgl1*^+/+ ^and *Lgl1*^+/- ^mice was quantified by quantitative real-time RT PCR (N ≥ 4). A significant reduction in tropoelastin mRNA was observed at E14.5 in the *Lgl1*^+/- ^mice. From E16.5 until PN1 tropoelastin mRNA levels in lungs of *Lgl1*^+/- ^mice were similar to those observed in wild type littermates (p < 0.05). At PN28, tropoelastin mRNA levels were significantly elevated relative to controls (p < 0.01).

To clarify the significance of the observed reduction of *Lgl1 *expression in mature mouse lung, we assessed lung function in *Lgl1*^+/+ ^and *Lgl1*^+/- ^mice at PN28. Initially, baseline lung mechanics were analyzed using the flexiVent small animal ventilator (Figure 5). No significant changes in lung resistance (*R*), compliance (*C*) or elastance (E) were observed when *Lgl1*^+/- ^mice were compared with *Lgl1*^+/+^littermates. We next administered methacholine (MCh), a smooth muscle agonist, to assess the effects on *R *and *E *of transient bronchoconstriction. Interestingly, increasing doses of MCh provoked a significantly *greater *increase in airway resistance (Figure 5A) and elastance (Figure 5B) in wild type mice than that observed in *Lgl1*^+/- ^mice. The observed effects on lung elastance suggested the possibility of altered elastin expression and/or deposition in developing lungs of *Lgl1*^+/- ^mice.

### Lungs of Lgl1^+/- ^mice display altered tropoelastin expression and disorganized elastin fibers

Chronic lung injury in the newborn has been associated with disordered elastin deposition[14]. Reduced lung elastance is also a characteristic feature of emphysematous lung[15]. Organization of the complex pulmonary elastin network is initiated in the pseudoglandular lung and peaks during alveolarization [16,17] Elastin deposition by myofibroblasts in late gestation is believed to have a spatially instructive role in alveolarization as specific sites of elastic fiber formation correspond to the location of future buds[18]. Elastin synthesis is initiated by expression of tropoelastin. To assess whether observed effects on lung elastance in *Lgl1*^+/- ^mice were associated with altered elastin synthesis, we measured tropoelastin mRNA (Figure 5C). A significant reduction in lung tropoelastin expression was observed at E14.5. From E16.5 until PN1 no significant differences in tropoelastin mRNA were detected until maturity. Interestingly, at PN28, concomitant with the observed reduction in *Lgl1 *expression, there was a significant increase in expression of tropoelastin mRNA (Figure 5C).

**Figure 6:**
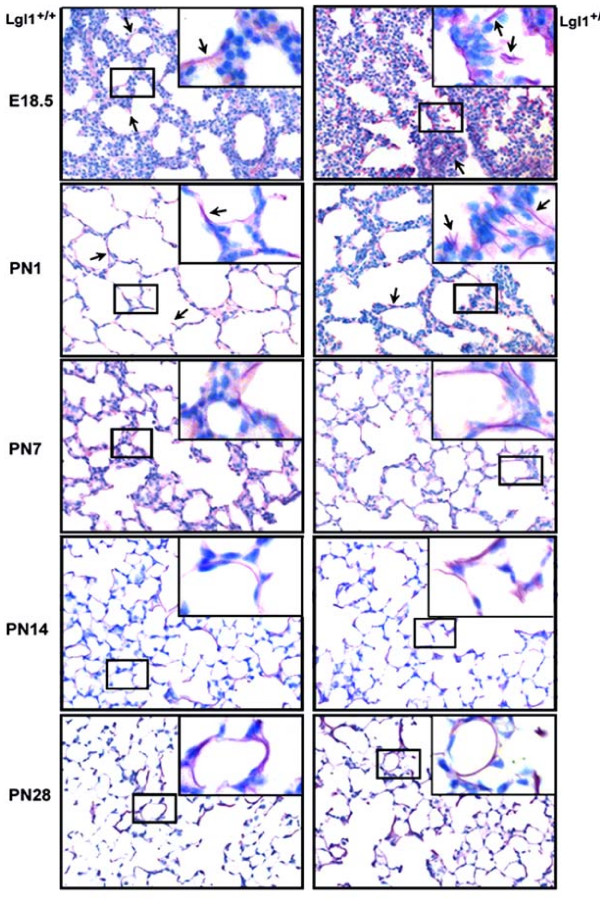
**Lgl1^+/- ^mice display disorganization of elastin fibers at E18.5 and PN1**. Lung sections of *Lgl1*^+/+ ^and *Lgl1*^+/- ^mice were treated with Weigert's elastin stain to visualize elastin fibres. Representative images are shown. In wild type mice, elastin fibres run longitudinally along the alveolar walls of lungs and protrude into the airspaces at the sites of secondary septal crests (see arrows). By contrast, at E18.5 and PN1 lungs of *Lgl1*^+/- ^mice show disorganization and fragmentation of elastin fibres (See also insets). Effects on elastin structure were less pronounced from PN7-PN14 and resolved at maturity. Magnification: 400×, insets: 1000×.

We next used Weigert's elastin stain to visualize elastin fibers in the lungs of *Lgl1*^+/- ^and wild type *Lgl1*^+/+ ^mice. Representative images are shown in Figure 6 (n > 4). At E18.5, elastin fibres in *Lgl1*^+/+ ^mice run longitudinally along the alveolar walls and protrude into the airspaces at the sites of secondary septal crests (Figure 6, see arrows). By contrast, elastin fibres in the lungs of *Lgl1*^+/- ^mice appeared disorganized and fragmented with trapping in the interstitium (See insets, Figure 6). At PN1 trapping of elastin fragments in the interstitium was still apparent. Moreover, less elastin was observed at sites of septal crests. At PN7 and PN14 effects on elastin distribution were much less pronounced. By PN28, these effects on elastin appeared to be resolved and elastin structure looked normal despite the presence of elevated levels of tropoelastin mRNA.

### Post-natal Lgl1^+/- ^mice display goblet cell hyperplasia

**Figure 7:**
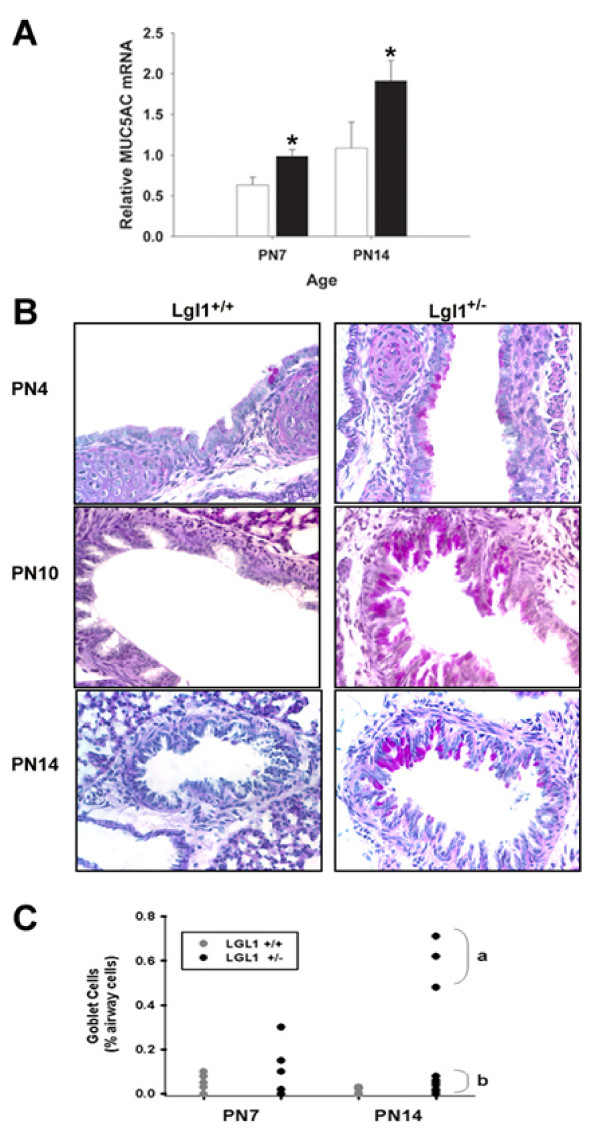
**Postnatal Lgl1^+/- ^mice display goblet cell hyperplasia and increased expression of MUC5AC**. A. MUC5AC was quantified in mRNA isolated from total lungs of *Lgl1*^+/+ ^and *Lgl1*^+/- ^mice by quantitative real-time RT PCR (N ≥ 5). A significant increase in MUC5AC mRNA was observed at PN7 and PN14 in the *Lgl1*^+/- ^mice (p < 0.05). B. Lung sections of *Lgl1*^+/+ ^and *Lgl1*^+/- ^mice were stained with Period Acid Schiff (PAS) stain to visualize the goblet cells. Representative images are shown.PAS positive cells were rarely seen in wild type mice during the early post natal period, however a considerable number of PAS positive cells were observed in both the trachea and upper bronchi of the *Lgl1*^+/- ^mice during this same time period. Magnification: 400× C. Scatter plot illustrating bimodal distribution of goblet cells in heterozygous *Lgl1*^+/- ^mice (significant difference between subgroups, p = 0.017, Mann Whitney U test).

Increased numbers of mucin-positive goblet cells are a characteristic feature of inflammation in multiple respiratory disease states including BPD. In order to determine whether observed changes in *Lgl1 *expression caused abnormalities in airway epithelial cells in the trachea and bronchi, lungs of *Lgl1*^+/- ^and *Lgl1*^+/+ ^mice were stained for mucin-positive goblet cells (PAS stain). Representative images are shown in Figure 7B (n ≥ 6). Whereas PAS positive cells were rarely seen in wild type mice from PN4 - PN14, a considerable number of mucin-positive goblet cells were observed in both the bronchi and trachea of a subset *Lgl1*^+/- ^mice during this period (Figure 7B). At PN14, *Lgl1*^+/- ^mice fell into two very distinct subgroups based on goblet cell number, those with an increased number of goblet cells and those that were not distinguishable from wild type littermates (significant difference between subgroups, p = 0.017, Mann Whitney U test; See scatter plot, Figure 7C). In no case were goblet cells elevated in wild type pups.

In the lungs, goblet cell hyperplasia of surface epithelial cells in inflammatory disease generally correlates with increased expression of mucin (MUC5AC) mRNA[19]. We therefore assessed MUC5AC mRNA expression in lungs of *Lgl1*^+/- ^and *Lgl1*^+/+ ^mice. Increased staining of goblet cells in PN lung of *Lgl1*^+/- ^mice was accompanied by an increase in expression of mucin (MUC5AC) mRNA (Figure 7A).

### Inflammatory cytokines, IL-4 and IL-13 are elevated at PN7 in Lgl1^+/- ^mice

**Figure 8:**
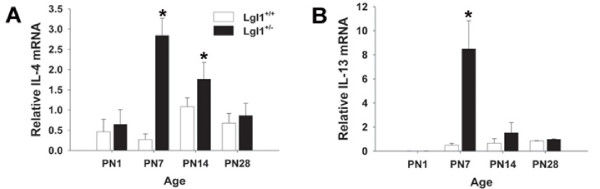
**Postnatal Lgl1^+/- ^mice display increased expression of IL-4 and IL-13 mRNA**. IL-4 and IL-13 mRNA was quantified in mRNA isolated from total lungs of *Lgl1*^+/+ ^and *Lgl1*^+/- ^mice by quantitative real-time RT PCR (N ≥ 4). A. *Lgl1*^+/- ^display significantly elevated IL-4 levels at PN7 and PN14 compared to wild type controls (p ≤ 0.05). B. *Lgl1*^+/- ^mice display significantly elevated levels of IL-13 at PN7 compared to wild type controls (p ≤ 0.05).

Inflammatory cytokines stimulate MUC5AC expression. The findings of altered mucin expression and goblet cell hyperplasia in PN lungs of *Lgl1*^+/- ^mice led us to ask whether *Lgl1 *may have a role in the development of immune modulation. We assessed levels of two inflammatory cytokines, IL-4 and IL-13. Dramatically elevated levels of both IL-4 and IL-13 were observed at PN7 (Figure 8). At PN14 IL-4 remained significantly elevated. The increase in IL-13 expression was no longer significant at this time. To determine whether elevated cytokine levels were associated with induction of recruitment of inflammatory cells, BAL cell differentials were determined at PN10 and at maturity. No significant differences in BAL cell counts were detected when *Lgl1+/- *mice were compared to wild type littermates.

## Discussion

The finding that null mutation of the *Lgl1 *gene in mouse embryos is lethal prior to the onset of lung morphogenesis classifies *Lgl1 *as an essential early gene in organismal development. The determinants of embryonic lethality in *Lgl1*^+/- ^mice remain to be determined and pre-date lung organogenesis. Mutation in *Lgl1 *has pleiotropic effects. Moreover, it is possible that effects on *Lgl1 *expression in other organ systems contribute to the postnatal phenotype observed in the lung.

The present study demonstrates that the heterozygous *Lgl1 *genotype is sufficient to induce a complex phenotype. In postnatal lung, histologically immature areas with distinctly thickened respiratory interstitium and the appearance of delayed secondary septation alternate with areas of relatively normal appearance. Disorganized elastin fibers, goblet cell hyperplasia and high levels of inflammatory cytokines are present in the absence of detectible differences in *Lgl1 *mRNA levels. Our inability to detect changes in *Lgl1 *mRNA expression in total RNA isolated from developing lung is likely to reflect a specific requirement for *Lgl1 *in a subpopulation of cells in which suppressed expression of *Lgl1 *is sufficient to produce the observed phenotype but escapes detection by RT-PCR of total lung RNA. For example, we showed previously that *Lgl1 *is maximally expressed in fibroblasts adjacent to the epithelium [1,2]. We also demonstrated that *Lgl1 *is secreted and taken up by epithelial cells beginning in late gestation and continuing in PN life [2]. Deficient *Lgl1 *expression in a subset of fibroblasts important in mesenchymal-epithelial interactions that regulate alveolarization may contribute to the observed phenotype.

We have backcrossed our *Lgl1 *^+/- ^mice onto the C57BL/6 background (eight generations). Neonatal *Lgl1 *^+/- ^mice on this background faithfully reproduce the phenotype of disorganized elastin, goblet cell hyperplasia with elevated levels of MUC5AC and increased IL-4 and IL-13 expression, demonstrating that the phenotype we report is not due to a mixed genetic background.

The association of a distinct respiratory phenotype in the absence of significant reduction in mRNA has been reported previously [13,20]. Foxf1 heterozygotes can be divided on the basis of pulmonary levels of Foxf1 mRNA[13]. Low Foxf1 producers fail to undergo differentiation of terminal airspaces. An *albeit less-severe defect in septation *of the lung periphery is *observed *in High Foxf1 1 producing heterozygotes mice that express normal or nearly normal (90%) levels of Foxf1 mRNA[13]. Moreover, these animals display aberrant expression of a number of developmentally important genes in the lung.

There was considerable variability in *Lgl1 *protein levels in *Lgl1*^+/- ^mice. No such variability was observed in wild type animals. We demonstrated regional differences in tissue fraction in postnatal *Lgl1*^+/- ^mice. Given that *Lgl1 *is of mesenchymal origin and that mesenchymal thinning is a prominent feature of lung maturation, variability in *Lgl1 *protein levels may reflect varying degrees of developmental delay. The observed reduction in *Lgl1 *protein in the lungs of *Lgl1*^+/- ^mice may also reflect effects on RNA stability or protein turnover Absence of *Lgl1 *in endothelial cells suggests that *Lgl1 *does not have a direct role in PN angiogenesis.

Genetic modifiers of tissue- and temporal-specific expression of *Lgl1 *may also contribute to the observed variation in penetrance of the *Lgl1*^+/- ^phenotype. In this context it is important to recall that low levels of *Lgl1 *in pseudoglandular lung are essential to airway branching[12]. Haplosufficiency for *Lgl1 *is clearly sufficient to rescue the branching program. At the same time, effects on tropoelastin expression were observed at E14.5 suggesting that the heterozygous phenotype does impact lung development in the pseudoglandular period.

Reduced *Lgl1 *expression in mature *Lgl1*^+/- ^mice was associated with normal baseline lung function. As expected, MCh challenge provoked an increase in airway resistance and elastance in both *Lgl1*^+/- ^and wild type littermates. However, the effects on both these parameters were significantly greater in wild type mice. Moreover, tropoelastin expression in mature *Lgl1*^+/- ^mice was elevated. Elastic interdependence of the lung accounts for orderly elastic recoil of lungs during passive expiration [21]. Organization of the elastin network is initiated in the pseudoglandular lung and peaks during alveolarization [16,17]. At the alveolar level, elastic interdependence is mediated by the correct expression, cross-linking, and orientation of elastin and collagen fibers. Absence of a correctly cross-linked and oriented elastin matrix predisposes to aberrant alveolarization. Deficient alveolar maturation in BPD includes disruption of elastin fibers, distal airspace enlargement, and mucus cell hyperplasia [14]. All of these properties were present in newborn *Lgl1*^+/- ^mice. Dysregulation of elastin synthesis is also a prominent feature of BPD in murine [22] and preterm lamb models [23].

Excessive degradation of the elastin matrix underlies the loss of elastic interdependence and alveolar degeneration associated with chronic lung disease in adults. Indeed, it is believed that individuals with even limited elastin insufficiency may suffer damage sufficient to preclude alveolar repair from a less severe injury than would be required to have this effect in normal subjects. Survivors of preterm birth are at particular risk to develop premature COPD [21,24]. The disrupted elastin architecture in *Lgl1*^+/- ^mice appears to resolve at maturity. Yet these animals have elevated tropoelastin levels and impaired lung function at maturity. It is tempting to speculate that latent effects on elastin integrity may increase vulnerability of *Lgl1*^+/- ^mice to respiratory insult at maturity and that *Lgl1 *haploinsufficiency may modify risk to respiratory insult in later life.

Given that the disruption of elastin expression and/or organization has been associated with lung injury in the newborn period, it was of interest to establish whether *Lgl1*^+/- ^mice displayed any other characteristic features associated with neonatal lung injury. Inflammation is known to interfere with lung development in model systems and is present chronically in the lungs of preterm infants who develop BPD [25]. Goblet cell hyperplasia is associated with multiple respiratory disorders including asthma, BPD and emphysema. It has been suggested that exposure of the developing lung to inflammation may be central to the development of BPD[25]. Analysis of goblet cell number in PN14 lungs of *Lgl1*^+/- ^mice identified a bimodal distribution, with one subgroup of animals displaying significantly elevated numbers of goblet cells in trachea and bronchi associated with elevated mucin (MUC5AC) production. A second group of animals showed no increase in goblet cell number. The presence of these 2 distinct groups is likely to reflect the incomplete penetrance of the *Lgl1*^+/- ^phenotype and may be attributed to the genetic contribution from C57BL/6 and 129/J strains. Indeed, we have found that on the C57BL6 background, goblet cell hyperplasia in *Lgl1*^+/- ^mice is much less variable.

The differentiation of epithelial cells to goblet cells is induced by inflammatory cytokines [19]. We found dramatically elevated levels of the T_H_2 cytokines IL-4 and IL-13 in the postnatal *Lgl1*^+/- ^mouse lung, concordant with evidence of goblet cell hyperplasia. Both IL-4 and IL-13 have been shown to induce goblet cell hyperplasia and mucus hypersecretion in mouse airways [26-28]. It is of interest that despite the pronounced rise in IL-4 and IL-13 observed at PN7, we did not detect an inflammatory infiltrate in BAL. Both Il-4 and IL-13 are cytokines associated with the inflammatory process in diseases such as asthma, but neither of these cytokines has chemokine properties. Indeed, Wills-Karp et al. [28] have shown that these cytokines can induce pathophysiological features of asthma via mechanisms independent of eosinophil recruitment. Thus the inflammation observed in postnatal *Lgl1*^+/- ^mice that are free of allergen and infection is un likely to involve recruitment of cellular infiltrates. The combined findings of goblet cell hyperplasia and induction of inflammatory cytokines are nevertheless consistent with a role for *Lgl1 *in innate immunity suggesting that *Lgl1 *may function to protect the lung from external insults.

To date, the domain structure of *Lgl1 *has offered little to our understanding of its molecular function. The LCCL domain family now includes more than 100 proteins. Orthologues of *Lgl1 *are typified by having two LCCL modules. Multiple functions have been attributed to the LCCL module (also known as FCH[5]). These include roles in cell differentiation, motility and migration [5,7]; cell adhesion and matrix deposition [8,9]; and host-defense and innate immunity [4,10,11]. The results of our studies provide the first evidence for a potential role of *Lgl1 *in innate immunity. Mutagenesis of the LCCL modules in *Lgl1 *will be necessary to explore such effects *in vitro*.

There are a number of limitations to this study. While haploinsufficiency for *Lgl1 *is the only reasonable explanation for the pulmonary phenotype in *Lgl1*^+/- ^heterozygotes, we were not able to identify the precise time and localization of deficient *Lgl1 *that triggers the postnatal phenotype. There was considerable variability in several outcome measures among *Lgl1*^+/- ^heterozygotes. The development of a mouse model with conditionally regulatable expression of *Lgl1 *will allow a more definitive analysis of the role of *Lgl1 *in early lung development.

## Conclusion

Absence of *Lgl1 *in null mice is embryonic lethal, making *Lgl1 *an essential gene. Neonatal *Lgl1*^+/- ^mice exhibit increased interstitial tissue, goblet cell hyperplasia and elevated cytokine levels. The disorganized elastin architecture seen in neonatal *Lgl1*^+/- ^mice would be expected to interfere with normal elastic recoil and may increase vulnerability to alveolar degeneration. Indeed, in adult *Lgl1*^+/- ^mice, which express 50% *Lgl1 *mRNA, reduced lung elastance is associated with elevated tropoelastin levels. Mice with *Lgl1 *haploinsufficiency display changes in lung phenotype that resemble those seen in chronic neonatal lung disease.

## Competing interests

The authors declare that they have no competing interests.

## Authors' contributions

JL generated the knockout mouse. LR and IM characterized *Lgl1 *mRNA and protein, elastin and tropoelastin mRNA, and goblet cells and mucin mRNA in *Lgl1*^+/- ^heterozygotes and wild type controls. TB participated in H and E histochemistry and protein quantitation. KN and SC carried out lung function studies. NBS contributed to the design of the study and the preparation of the manuscript. FK conceived and participated in the design of the study and had a primary role in preparation of the manuscript.

## Supplementary Material

Additional file 1 **Generation of *Lgl1 *KO Mouse**. Detailed description of the generation of the *Lgl1 *KO mouse Click here for file

Additional file 2 **Additional methods**. Detailed explanation of methods used for *Lgl1 *immunohistochemistry, quantitative real-time PCR and pulmonary function studies. Click here for file
