# Supplementary material for: Inflammatory cytokines, goblet cell hyperplasia and altered lung mechanics in Lgl1+/- mice
Source: Respir Res. 2009 Sep 21;10(1):83. doi: 10.1186/1465-9921-10-83 (PMC2760518; doi:10.1186/1465-9921-10-83)
Supplement: Additional file 1 — Generation of Lgl1 KO Mouse. Detailed description of the generation of the Lgl1 KO mouse [file 1465-9921-10-83-S1.DOC]

# Additional file 1

**File format**: DOC

**Title**: Generation of *Lgl1* KO Mouse

**Description**: Detailed description of the generation of the *Lgl1* KO mouse

A 13.7kb EcoRI fragment of BAC clone 34304 was prepared containing the entire genomic sequence of *Lgl1.* *Lgl1* exons 2-6 were cloned into the EcoRI site of pQZ1BamHI (a gift of Dr. Qin-zhang Zhu, Institute de Recherche Clinique de Montreal, Montreal). The clone was verified by *in-situ* hybridization using a 600bp *Lgl1* genomic probe, spanning the region between exons 2 and 3, prepared by PCR amplification (Right primer: 5’-TCAGGAGCCTACCCAAGAACTC TGC; Left primer: 5’-AGCAGGTGGGGGAGG AAAGAG TTTA). Exon 2 was removed by

Hind III digestion. The DNA fragment was treated with Klenow, and then partially digested to produce a 12.7kb fragment with one blunt-end and one BamHI end. A Neo gene cassette with the same ends (plasmid pMC1Neo PolyA, digested with XhoI, filled with Klenow, then digested with BamHI) was then cloned into the vector. Accurately targeted clones were identified by *in-situ* hybridization with a 1.1kb Neo gene fragment. Finally, the plasmid construct was digested with NotI and SalI to generate a 9kb targeting fragment that contains *Lgl1* exon3-6 with exon 2 replaced by the Neo gene.

**ES cell culture and micro-injection**

ES cells (129/Sv x 129/Sv-CP, F1 3.5-day blastocyst) were purchased from Dr. Andras Nagy (Mount Sinai Hospital, Toronto, ON). 5x10^7^ cells were resuspended in 0.8mL DMEM and electroporated with 25 μg purified DNA targeting fragment in PBS using Gene Pulser (BioRad Cat#165-2098) at 220v, 500-900μF. ES cells were then subjected to G418 (Invitrogen Cat #11811-031) (220 μg/mL final concentration) selection for Neo resistant clones. Six hundred G418 resistant clones were selected and subjected to Southern analysis and PCR screening. A single clone 12G was chosen for micro-injection into C57Bl1 mice blastocysts.

**Genotyping heterozygotes and wild-type KO mice**

Chimeric mice were bred to generate *Lgl1^+/-^* heterozygotes. Mouse genotypes were verified by PCR of tail DNA. The primers used were (5’-3’): reverse(wild type) CACTGCTCCGTGTATC AAGCATACAC; reverse (NeoI) GACAATCG GCTGCTCTGATG; or reverse (5' to3') TCGTCGTGACCCATGGCGAT (NeoII) and forward (for all 3 reactions) CAGGTCTGGCTCTGAGGTTCTTGCA. The expected amplification products were: 0.8kb (wild type), 1kb (Neo1) and 0.46kb (Neo2).

### Results

*Absence of lgl1 is associated with embryonic lethality –* An *Lgl1* knockout mouse was generated by introduction of a neomycin cassette in exon 2 of the *Lgl1* gene (Figure 1A). Accuracy of the targeting in ES cells was confirmed by Southern analysis using both 5’ and 3’ probes. The 3’ probe identifies two expected *Lgl1* fragments (13.7kb [wild type], 8.4kb [mutant]) following EcoR1 digestion (Figure 1B). The 5’ Neo cassette probe identifies a 5.5 kb fragment following HindIII digestion (Figure 1B). Mouse genotypes were verified by PCR using two sets of mutant-specific primers, generating respectively, 0.8kb, 1kb and 0.46kb fragments for the wild type and 2 mutant amplification products (Figure 1C).

**
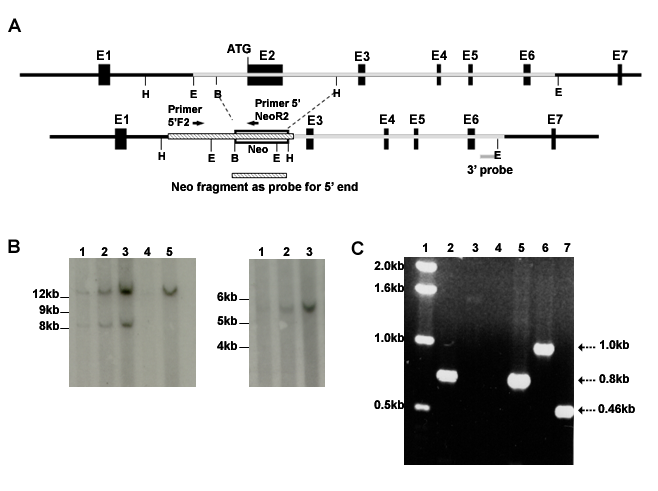
**
