# Supplementary material for: Inflammatory cytokines, goblet cell hyperplasia and altered lung mechanics in Lgl1+/- mice
Source: Respir Res. 2009 Sep 21;10(1):83. doi: 10.1186/1465-9921-10-83 (PMC2760518; doi:10.1186/1465-9921-10-83)
Supplement: Additional file 2 — Additional methods. Detailed explanation of methods used for Lgl1 immunohistochemistry, quantitative real-time PCR and pulmonary function studies. [file 1465-9921-10-83-S2.DOC]

# =

# Additional file 2

**File format**: DOC

**Title**: Additional methods

**Description**: Detailed explanation of methods used for *Lgl1* immunohistochemistry, quantitative real-time PCR and pulmonary function studies.

***Lgl1* Immunohistochemistry**

Perfused lung tissue sections (n>4 animals/group) were inflation-fixed and prepared for immunohistochemistry using the avidin-biotin-peroxidase technique as described ^1^. A rabbit polyclonal lgl1 antibody was raised against two synthetic lgl1 peptides ^2^ by Medicorp (Montreal, QC, Canada). The antibody identifies the expected protein band at 52kD. Confirmation of the lgl1 target was previously demonstrated by preabsorption of the antibody with 5-fold excess of each peptide, which eliminated lgl1 staining ^2^. The primary antiserum was diluted 1:100 and left overnight at room temperature. Antibody specificity was verified by omitting the primary antibody. The following day a corresponding fluorescent conjugated secondary antibody was added for 30 minutes at room temperature. After immunostaining, digital images were taken using a Spot camera (Diagnostic).

**Quantitative real-time PCR.**

Real-time/Quantitative RT-PCR was performed on the Mx4000 QPCR system from Stratagene (Stratagene, La Jolla, CA) using the Quantitect One-Step Probe RT-PCR Kit (Qiagen). Gene-specific primers and FAM-labelled probes for mouse *Lgl1*, were designed using Qiagen’s online QuatiProbe Design Software. Quantitect Gene Expression Assay for mouse 18S (Qiagen) was used to normalize for the input of RNA. The results were analyzed according to the standard curve method^3^. One-step real-time RT-PCR reactions were performed in 25μL volumes for 45 cycles, using 20ng of total RNA for *Lgl1*, IL-4, IL-13, Mucin5AC and Tropoelastin and 50pg for 18S. Primers and probes used are outlined in Tables S1 and S2.

**Pulmonary function studies** - Lung mechanics were assessed in 28 day old *Lgl1^+/-^* and wild type male mice (n>13) using a computerized flexiVent system (Scireq) as described^4^*.* Mice were weighed and then deeply anesthetized by an intraperitoneal injection of xylazine (8 mg/kg) and pentobarbital (70 mg/kg), tracheostomized, and connected to a computer-controlled small animal ventilator (flexiVent; SCIREQ, Montreal, PQ, Canada). Regular quasisinusoidal ventilation was delivered at a frequency of 150 breaths / minute with a tidal volume of 10 mL/kg at positive end-expiratory pressure (PEEP) level of 3 cm H_2_O. The mouse was paralyzed with pancuronium bromide (0.8 mg/kg intraperitoneally), and after a standard volume history, a “standardized breath” type signal at 150 breaths/min (2.5 Hz) was applied to measure the single compartment model parameters (resistance; R, compliance, C and elastance, E). This measurement was followed by 1 minute of regular ventilation and then an 8 second broadband signal to measure input impedance from 0.5 to 19.75 Hz was delivered. The resulting impedance was further analyzed using the constant-phase model to obtain the parameters for resistance (Rn), elastance (G) and compliance (H).

**References**

1. Han, R. N., S. Buch, I. Tseu, J. Young, N. A. Christie, H. Frndova, S. J. Lye, M. Post, and A. K. Tanswell. 1996. Changes in structure, mechanics, and insulin-like growth factor-related gene expression in the lungs of newborn rats exposed to air or 60% oxygen. *Pediatr.Res.* 39:921-929.

2. Oyewumi, L., F. Kaplan, S. Gagnon, and N. B. Sweezey. 2003. Antisense Oligodeoxynucleotides Decrease LGL1 mRNA and Protein Levels and Inhibit Branching Morphogenesis in Fetal Rat Lung. *Am J Respir Cell Mol Biol* 28:232-240.

3. Bustin, S. A. 2002. Quantification of mRNA using real-time reverse transcription PCR (RT-PCR): trends and problems. *J Mol Endocrinol* 29:23-29.

4. Schuessler, T. F., J. H. Bates, T. F. Schuessler, and J. H. Bates. 1995. A computer-controlled research ventilator for small animals: design and evaluation. *IEEE Transactions on Biomedical Engineering* 42:860-866.

**Table S1.** Gene-specific primers and FAM-labelled probes

| **Gene** | **Primers** | **Probe** |
| --- | --- | --- |
| **Lgl1** | 5’-CCCTTCTTTGTCAAGTCTC-3’  5’-GCCGTCTCTGTCACTTTT-3’ | 5’-CCGTCTAGCTCCTTCACT-3’ |
| **MUC5AC** | 5’-CAGCGTGGAGAATGAAAAGTAT-3’ 5’-ATGCAGTTCGAGAAGAAGGTG-3’ | 5’-TGGTGTTCTCTGCTGAC-3’ |
| **TROPO ELASTIN** | 5’-CCCATCAAAGCACCAAA-3’  5’-CCTGCACCAGCTACTCCATA-3’ | 5’-GGACTGCCCTATACCA-3’ |
| **SP-A** | 5’-GCAGTGTGATTGGGAGAA-3’  5’-CAGCAACAACAGTCAAGAAGA-3’ | 5’-TGTCACTAGGCTCTTTGG-3’ |
| **SP-D** | 5’-CAACAAGGAAGCAATCTGACAT-3’  5’-CAGGTGTTGGGTACTGATCTCT-3’ | 5’-CTCTCCATGCTTGTCTT-3’ |
| **IL-4** | 5’-GAGATCATCGGCCATTTTG-3’  5’-TCACTCTCTGTGGTGTTCTTC-3’ | 5’-CGGAGATGGATGTGCCAA-3’ |
| **IL-13** | 5’-CATCACACAAGACCAGACTC-3’  5’-GAATCCAGGGCTACACAGAA-3’ | 5’-CAACGGCAGCATGGTA-3’ |

**Table S2.** Primers for genotyping PCR – Forward primer is the same for all primer sets

| **Primer** | **Sequence** |
| --- | --- |
| **Wildtype** | F: 5’-CACTGCTCCGTGTATCAAGCATACAC-3’  R: 5’-CAGGTCTGGCTCTGAGGTTCTTGCA-3’ |
| **Neo I** | R: 5’-GACAATCGGCTGCTCTGATG-3’ |
| **Neo II** | R: 5’-TCGTCGTGACCCATGGCGAT-3’ |
